# Supplementary material for: Atropos: specific, sensitive, and speedy trimming of sequencing reads
Source: PeerJ. 2017 Aug 30;5:e3720. doi: 10.7717/peerj.3720 (PMC5581536; doi:10.7717/peerj.3720)
Supplement: Supplemental Information 1 [file peerj-05-3720-s001.pdf]

Supplement to *Atropos: specific, sensitive, and speedy trimming of sequencing reads*

John P Didion[1], Marcel Martin[2], and Francis S Collins[1]

[1]National Human Genome Research Institute, National Institutes of Health, Bethesda, MD
[2]Science for Life Laboratory, Department of Biochemistry and Biophysics, Stockholm University, Sweden

Corresponding author:
John P Didion, PhD[1]

Email address: john.didion@nih.gov

1 FIGURES

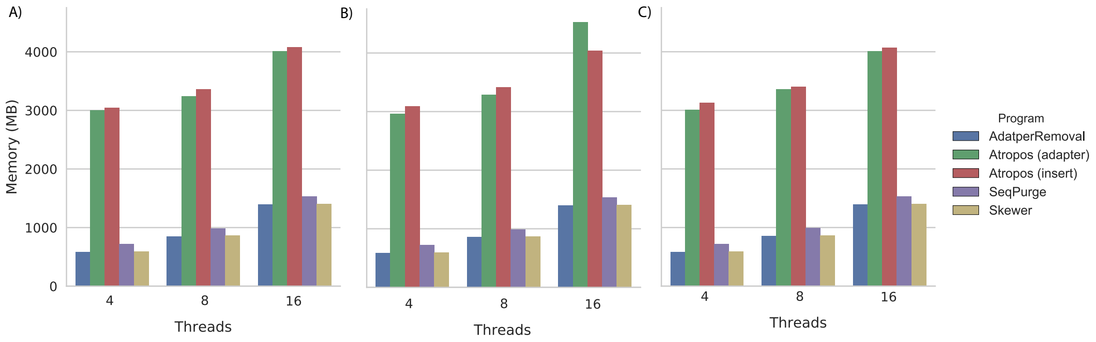

Figure 1. Memory usage of trimming tools on simulated datasets. Maximum memory usage, in MB, of jobs executed on our cluster for trimming tools run on simulated datasets with error rates of A) 0.2%, B) 0.6%, and C) 1.2%. Note that this memory usage includes the overhead of the Singularity container and is thus an overestimate.

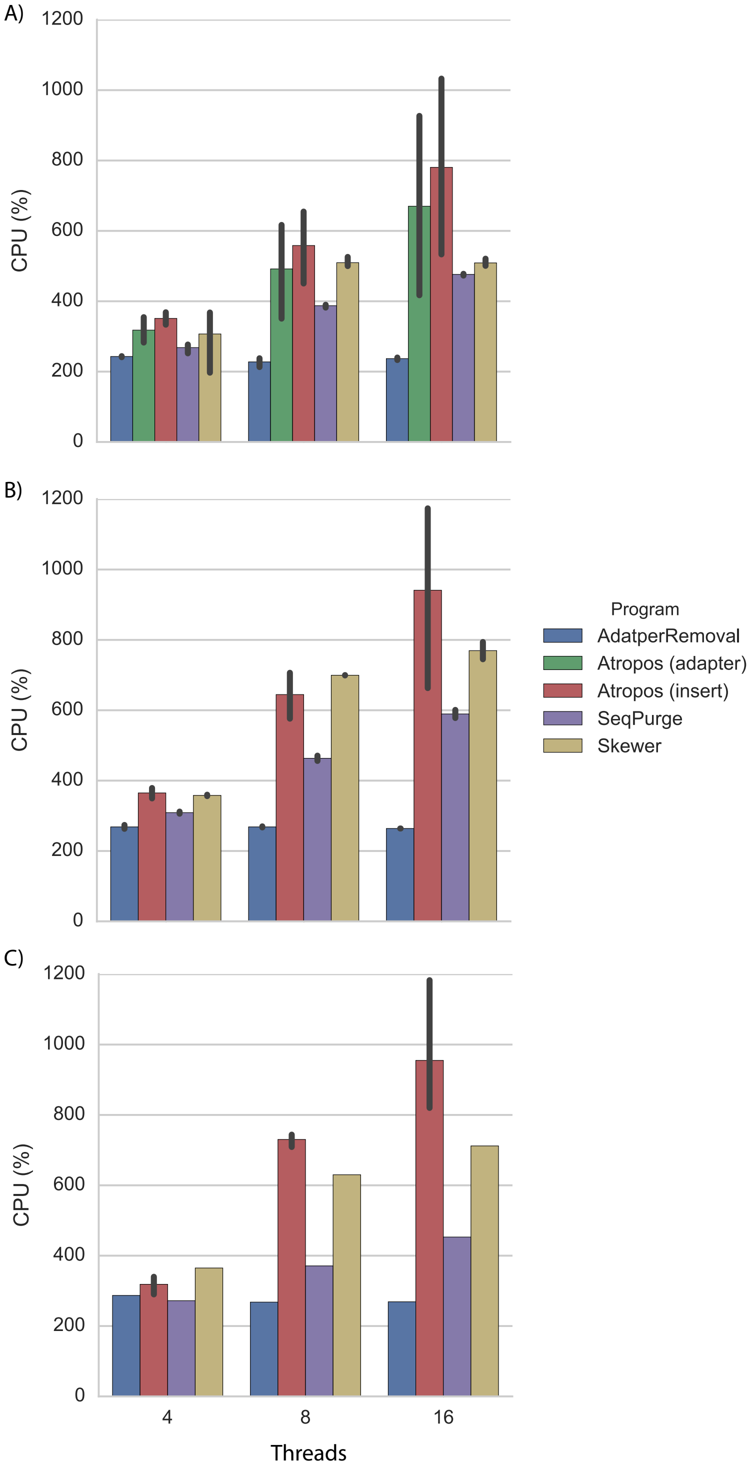

Figure 2. CPU Utilization of trimming tools. Average total CPU usage of each trimming tool run on A) simulated data, B) WGBS data, and C) mRNA-Seq data.

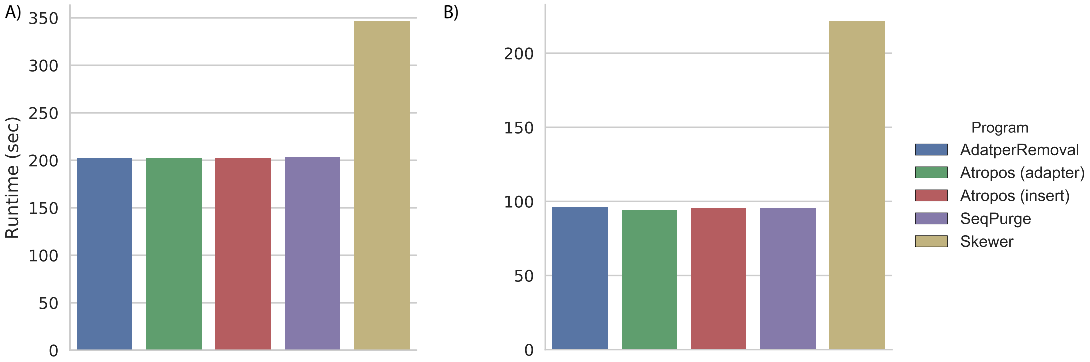

Figure 3. Mapping execution times. Execution time of A) bwa-meth on WGBS reads, and B) STAR on mRNA-Seq reads, for reads trimmed by each tool as well as the untrimmed reads.

2 TABLES

Software	Version	Citation
**Trimmers**		
Atropos	1.1.5	This manuscript
AdapterRemoval	2.2.0	Schubert et al. (2016)
SeqPurge	ngs-bits commit 9e8d99d	Sturm et al. (2016)
Skewer	0.2.2	Jiang et al. (2014)
**Aligners**		
BWA	0.7.15	Li (2013)
bwa-meth	commit 1c530af	Pedersen et al. (2014)
Samtools	1.4	Li et al. (2009)
STAR	2.5.3a	Dobin et al. (2013)
**Misc**		
ART	151	Huang et al. (2012); Jiang et al. (2014)
Bedops	2.4.26	Neph et al. (2012)
SRA toolkit	2.8.2-1	`https://github.com/ncbi/sra-tools`

Table 1. Descriptions of software used in the benchmark workflow.

Program	Execution Time (sec.) Min	Max
AdapterRemoval	**20.31**	20.54
Atropos (adapter + nowriter)	32.72	43.45
Atropos (adapter + worker)	79.69	82.14
Atropos (adapter + writer)	47.54	52.34
Atropos (insert + nowriter)	42.21	49.19
Atropos (insert + worker)	90.11	96.02
Atropos (insert + writer)	49.7	55.01
SeqPurge	22.02	22.73
Skewer	39.03	43.28
	CPU Usage (%)	
AdapterRemoval	236	238
Atropos (adapter + nowriter)	322	332
Atropos (adapter + worker)	365	366
Atropos (adapter + writer)	223	239
Atropos (insert + nowriter)	338	360
Atropos (insert + worker)	371	372
Atropos (insert + writer)	291	294
SeqPurge	175	178
Skewer	250	259

Table 2. Min/max execution time and average CPU usage for trimming of simulated datasets on a desktop with 4 parallel threads.

	4 Threads		8 Threads		16 Threads	
Program	Execution Time (Min|Max sec.)					
AdapterRemoval	**24.05**	25.68	24.45	26.7	23.75	25.69
Atropos (adapter + nowriter)	39.15	49.45	**22.03**	31.71	**16.78**	23.9
Atropos (adapter + worker)	105.79	107.07	58.26	71.39	31.72	32.33
Atropos (adapter + writer)	54.93	56.59	49.59	56.82	44.61	58.79
Atropos (insert + nowriter)	53.17	60.92	31.72	40.2	19.7	30.59
Atropos (insert + worker)	120.15	154.89	63.5	81.31	35.3	36.22
Atropos (insert + writer)	61.4	78.8	51.71	61.7	45.56	50.66
SeqPurge	37.86	42.06	27.12	27.83	22.13	22.54
Skewer	34.37	65.44	29.44	32.52	29.66	31.69
	CPU Usage (Min|Max %)					
AdapterRemoval	241	244	213	238	233	240
Atropos (adapter + nowriter)	301	360	420	634	486	641
Atropos (adapter + worker)	372	375	665	709	1212	1228
Atropos (adapter + writer)	239	245	228	237	209	217
Atropos (insert + nowriter)	324	374	506	708	571	921
Atropos (insert + worker)	373	378	705	715	1229	1257
Atropos (insert + writer)	317	329	338	362	284	316
SeqPurge	252	277	382	390	473	478
Skewer	197	368	500	526	496	521

Table 3. Min/max execution time and average CPU usage for trimming of simulated datasets on a cluster node with 4, 8, or 16 parallel threads.

Program	16 Threads	4 Threads	8 Threads
Error rate 0.2%			
AdapterRemoval	1401.3	**589.1**	**854.6**
Atropos (adapter + nowriter)	**359.7**	2282.8	1676.2
Atropos (adapter + worker)	3893.3	3001.0	3241.2
Atropos (adapter + writer)	4015.4	2166.4	2231.8
Atropos (insert + nowriter)	1997.9	2532.0	1564.3
Atropos (insert + worker)	4078.1	3050.9	3366.3
Atropos (insert + writer)	4028.8	2538.4	2769.1
SeqPurge	1530.6	719.6	989.9
Skewer	1407.0	595.0	865.7
Error rate 0.6 %			
AdapterRemoval	**1397.6**	**584.8**	**857.5**
Atropos (adapter + nowriter)	4526.3	2395.9	2302.3
atropos (adapter + worker)	3702.2	2964.3	3282.6
Atropos (adapter + writer)	4013.4	2059.2	2211.6
Atropos (insert + nowriter)	3856.3	2383.7	2626.6
Atropos (insert + worker)	3847.8	3087.6	3413.9
Atropos (insert + writer)	4035.8	2557.0	2742.6
SeqPurge	1530.6	719.6	989.9
Skewer	1406.8	595.0	865.5
Error rate 1.2%			
AdapterRemoval	**1397.6**	**583.6**	**856.4**
Atropos (adapter + nowriter)	1998.0	2079.2	1234.5
Atropos (adapter + worker)	3785.6	3014.0	3362.4
Atropos (adapter + writer)	4011.3	2263.4	2227.9
Atropos (insert + nowriter)	1890.0	2343.2	1976.3
Atropos (insert + worker)	3530.3	3130.2	3405.0
Atropos (insert + writer)	4072.1	2487.2	2216.1
SeqPurge	1530.6	719.6	989.9
Skewer	1407.2	595.2	865.8

Table 4. Memory usage of jobs run on cluster for trimming simulated datasets.

	4 Threads		8 Threads		16 Threads	
Program	Execution Time (Min|Max sec.)					
AdapterRemoval	**30.79**	31.99	**22.0**	23.14	**23.19**	23.47
Atropos (insert + nowriter)	93.13	97.77	39.81	40.18	24.57	24.65
Atropos (insert + worker)	172.34	174.27	71.12	71.61	39.01	39.31
Atropos (insert + writer)	100.11	102.49	51.25	53.69	45.98	46.5
SeqPurge	60.09	60.77	37.07	37.76	30.24	31.18
Skewer	56.97	58.27	32.64	33.1	29.2	32.11
	CPU Usage (Min|Max %)					
AdapterRemoval	263	274	267	270	264	264
Atropos (insert + nowriter)	372	377	688	698	1003	1035
Atropos (insert + worker)	382	383	713	721	1299	1311
Atropos (insert + writer)	336	340	504	544	487	512
SeqPurge	306	312	456	471	578	601
Skewer	356	360	699	700	745	794

Table 5. Min/max execution time and average CPU usage for trimming of WGBS data on a cluster node with 4, 8, or 16 parallel threads.

	4 Threads	8 Threads	16 Threads
Program	Execution Time (sec.)		
AdapterRemoval	**102.0**	**98.91**	**99.71**
Atropos (insert + nowriter)	362.12	173.54	155.11
Atropos (insert + worker)	593.21	248.34	145.78
Atropos (insert + writer)	399.53	178.98	156.22
SeqPurge	232.65	178.67	140.44
Skewer	203.81	153.39	129.91
	CPU Usage (%)		
AdapterRemoval	287	268	269
Atropos (insert + nowriter)	340	744	862
Atropos (insert + worker)	326	709	1183
Atropos (insert + writer)	290	738	820
SeqPurge	272	371	453
Skewer	365	630	712

Table 6. Min/max execution time and average CPU usage for trimming of mRNA-Seq data on a cluster node with 4, 8, or 16 parallel threads.

REFERENCES

Dobin, A., Davis, C. A., Schlesinger, F., Drenkow, J., Zaleski, C., Jha, S., Batut, P., Chaisson, M., and Gingeras, T. R. (2013). STAR: ultrafast universal RNA-seq aligner. *Bioinformatics*, 29(1):15–21.

Huang, W., Li, L., Myers, J. R., and Marth, G. T. (2012). ART: a next-generation sequencing read simulator. *Bioinformatics*, 28(4):593–594.

Jiang, H., Lei, R., Ding, S.-W., and Zhu, S. (2014). Skewer: a fast and accurate adapter trimmer for next-generation sequencing paired-end reads. *BMC Bioinformatics*, 15:182–None.

Li, H. (2013). Aligning sequence reads, clone sequences and assembly contigs with BWA-MEM. *arXiv:1303.3997 [q-bio]*. arXiv: 1303.3997.

Li, H., Handsaker, B., Wysoker, A., Fennell, T., Ruan, J., Homer, N., Marth, G., Abecasis, G., and Durbin, R. (2009). The Sequence Alignment/Map format and SAMtools. *Bioinformatics*, 25(16):2078–2079.

Neph, S., Vierstra, J., Stergachis, A. B., Reynolds, A. P., Haugen, E., Vernot, B., Thurman, R. E., John, S., Sandstrom, R., Johnson, A. K., Maurano, M. T., Humbert, R., Rynes, E., Wang, H., Vong, S., Lee, K., Bates, D., Diegel, M., Roach, V., Dunn, D., Neri, J., Schafer, A., Hansen, R. S., Kutyavin, T., Giste, E., Weaver, M., Canfield, T., Sabo, P., Zhang, M., Balasundaram, G., Byron, R., MacCoss, M. J., Akey, J. M., Bender, M. A., Groudine, M., Kaul, R., and Stamatoyannopoulos, J. A. (2012). An expansive human regulatory lexicon encoded in transcription factor footprints. *Nature*, 489(7414):83–90. 00369.

Pedersen, B. S., Eyring, K., De, S., Yang, I. V., and Schwartz, D. A. (2014). Fast and accurate alignment of long bisulfite-seq reads. *arXiv:1401.1129 [q-bio]*. arXiv: 1401.1129.

Schubert, M., Lindgreen, S., and Orlando, L. (2016). AdapterRemoval v2: rapid adapter trimming, identification, and read merging. *BMC research notes*, 9:88.

Sturm, M., Schroeder, C., and Bauer, P. (2016). SeqPurge: highly-sensitive adapter trimming for paired-end NGS data. *BMC Bioinformatics*, 17:208.
